# Comparison of 3 vs 5 Fraction Single Isocenter Radiosurgery for Brain Metastases

**DOI:** 10.1016/j.adro.2025.101927

**Published:** 2025-10-23

**Authors:** Luke A. Moradi, Richard A. Popple, Roman L. Travis, Samuel R. Marcrom, Kristen O. Riley, James M. Markert, Christopher D. Willey, Michael C. Dobelbower, D. Hunter Boggs, Rodney J. Sullivan, Joel Pogue, John B. Fiveash

**Affiliations:** a Department of Radiation Oncology, University of Alabama at Birmingham, Birmingham, Alabama; b Department of Neurosurgery, University of Alabama at Birmingham, Birmingham, Alabama

## Abstract

**Purpose:**

Single isocenter stereotactic radiosurgery (SRS) efficiently delivers radiation to patients with multiple brain metastases. Although several fractionated SRS (fSRS) regimens show acceptable local control and toxicity, few studies directly compare them. This retrospective study evaluates 2 common regimens—6 Gy × 5 fractions and 9 Gy × 3 fractions—for their effects on local control and toxicity.

**Methods and Materials:**

A retrospective review was conducted of 1215 brain tumors from 251 patients receiving either 9 Gy × 3 fx or 6 Gy × 5 fx fSRS. All tumors were treated with single isocenter volumetric modulated arc therapy. Recurrent tumors and postoperative cavities were excluded from the analysis. Local tumor failure was defined as 25% increase in maximum tumor diameter (minimum 3 mm) or more than scant tumor cells at time of salvage surgery. Toxicity included CTCAE V5.0 central nervous system (CNS) grade 3 or greater events. Local tumor control and freedom from toxicity were calculated using Kaplan–Meier method and Cox regression models.

**Results:**

Overall local control was 93% at 1 year and 88% at 2 years. The 3-fraction regimen had superior 1-year local control compared with the 5-fraction regimen (97% vs. 91%, *P* = .001). Tumors <2 cm had significantly better control with 3 fractions (99% vs. 95%, *P* = .004), whereas tumors 2–4 cm showed no significant difference. One-year freedom from grade 3+ toxicity was similar between regimens (99% for 3-fx vs. 96% for 5-fx, *P* = .097).

**Conclusions:**

In this study, 9 Gy × 3 fx for brain metastases had improved tumor control and comparable toxicity to 6 Gy × 5, particularly among tumors <2 cm. 9 Gy × 3 fx may be the preferred regimen when treating multiple tumors with one prescription using single isocenter radiosurgery as it improves efficiency and local control while having similar toxicity.

## Introduction

It is estimated that 10%-40% of patients with solid tumors will develop brain metastases throughout the course of their treatment.^1^ Brain metastases cause significant morbidity and quality of life detriment, resulting in significant resource allocation toward treatment development. Historically, patients with brain metastases were offered whole brain radiation therapy (WBRT) regardless of number of metastases. Because WBRT has significant cognitive side effects, concerted efforts have been made to move toward focal therapy.^2^^,^^3^

With the development of the Gamma Knife and other forms of multiple isocenter radiosurgery, focal therapy was able to be offered for patients with moderate number of brain metastases.^4^ Unfortunately, treating each tumor sequentially carries several logistical challenges limiting the number of lesions eligible for treatment in a single course. More recently, the development of LINAC-based single isocenter stereotactic radiosurgery (SRS) with frameless single isocenter treatment planning improved many of the inefficiencies of early techniques, making it possible to treat many brain metastases throughout the brain in a matter of minutes.^5^, ^6^, ^7^, ^8^, ^9^ Development of LINAC-based SRS also increased access to care in rural communities because radiation oncology centers do not have to invest in a separate machine to specifically treat brain metastases.

Several dose regimens have proven effective and safe in treating brain metastases.^10^, ^11^, ^12^, ^13^, ^14^ The feared safety risk with SRS or fSRS is radionecrosis, which increases with dose and treatment volume. To simplify treatment planning for patients with multiple brain metastases, many centers use a single-dose prescription determined by the largest/most eloquently located tumor. Patients with smaller tumors (<2 cm) often are treated with single fraction SRS with doses of 18-22 Gray (Gy) to all tumors.^15^, ^16^, ^17^, ^18^ Patients with tumors >2 cm often require hypofractionated regimens (fSRS) to achieve high tumor control with acceptable risk of radionecrosis.^19^, ^20^, ^21^, ^22^, ^23^, ^24^ Regimens such as 9 Gy × 3 fraction (fx) or 6 Gy × 5 fx have been shown to be effective but little data exists comparing fSRS regimens.^10^^,^^23^ The purpose of this retrospective study is to compare 9 Gy × 3 fx (27 Gy) to 6 Gy × 5 fx (30 fx). We hypothesize that the higher biologically effective dose from 9 Gy × 3 fx will result in higher control but may also increase grade 3 or higher central nervous system (CNS) toxicity.

## Materials and Methods

A retrospective review was conducted of all patients with brain metastases who received 9 Gy × 3 fx (27 Gy) or 6 Gy × 5 fx (30 Gy) in our department from 2015 to 2022. Patients who underwent fSRS for previously untreated, intact brain metastases with minimum of one posttreatment diagnostic image (contrasted MRI or CT) were included in our analysis. Tumors that were recurrent, previously irradiated, or treated with a preoperative or postoperative intent were excluded. Brain metastases of lymphoma or germ cell histology were also excluded. In the case of multiple intracranial metastases and surgical resection of one tumor, the remaining intact tumors were included in our analysis of local control and toxicity. For patients who received prior WBRT, only tumors that had progressed since WBRT were included. Dural recurrences after resection that were marginal or include cavity were excluded whereas focal distant abnormalities involving the dura were included. This study was approved by the University of Alabama at Birmingham (UAB) Institutional Review Board for Human Use.

### Treatment

All cases of potential radiosurgery candidates underwent a multidisciplinary review. The decision to use fSRS was individualized and based on physician preference with an included institutional emphasis to use 3 fraction regimens during COVID. For patients with multiple brain metastases, the size of the largest lesion typically determined the fractionation schedule. In general, tumors >4 cm in diameter, near critical or eloquent structures, or tumors subject to dose bridging were treated 6 Gy × 5 fx (30 Gy). In contrast, patients with tumors <4 cm in diameter received 6 Gy × 5 fx (30 Gy) or 9 Gy × 3 fx (27 Gy) to all treated tumors per physician preference.

Patients were simulated in the supine position with an IziMed or Encompass device for immobilization. The gross tumor volume (GTV) was defined as enhancing abnormality identified on T1 postcontrast MRI sequence and CT scan. The total prescription dose was either 30 Gy delivered in 5 fx or 27 Gy delivered in 3 fx prescribed volumetrically such that at least 99% of the GTV received the entire prescription dose delivered over a 3 to 14-day period. No planning target volume margin was used.

All tumors were treated using fSRS with a single isocenter volumetric modulated arc therapy (VMAT) plan using Varian Eclipse (Varian Medical Systems). Treatment planning was performed manually (RapidArc) or via automated VMAT (HyperArc) using an 10× flattening filter free beam with no hotspot penalty. Daily patient alignment was confirmed via daily kV orthogonal radiographs followed by cone beam CT immediately prior to treatment. Surface imaging was used to detect intrafraction motion after 2016. Prior to that time, repeat cone beam CT was performed between arcs.

### Endpoints and statistical analysis

Local tumor failure was defined as 25% increase in maximum tumor diameter (minimum 3 mm) or more than scant tumor cells at time of salvage surgery. Local failure was graded on an individual tumor basis. Date of treatment failure was scored at first MRI that met size criteria listed above. For tumors that met size criteria then were subsequently resected, failure date was backdated to MRI that first met size criteria. Failure was also scored for growing lesions that did not meet size criteria but were retreated with second course of SRS because of physician judgment. Marginal dural failures within 5 mm were scored as failure for the original tumor. In cases of cerebral hemorrhage, distinct areas of hemorrhage separate from the tumor were excluded from tumor volume measurements. However, if the tumor and hemorrhage were indistinguishable, the entire lesion was included in the tumor volume.

Toxicity was defined using CTCAE V5.0 CNS grade 3 or greater events. Of note, CTCAE V5.0 differs from RTOG with the inclusion of “new onset seizures” as a grade 3 toxicity. Toxicity events were graded on an individual tumor basis. If causative tumor of toxicity event was unclear between 2 tumors, both tumors were assigned the toxicity event. In the event of tumor resection with pathology revealing extensive tumor necrosis, toxicity event was dated on day of resection. Resected tumors with elements of malignancy and extensive background necrosis on pathology were scored as both treatment failure and toxicity event. At time of tumor failure, “date of last toxicity check” was defined by the following: (1) if tumor was resected or re-irradiated, last toxicity check was date of retreatment, and (2) if tumor was retrospectively labeled as “failure” but observed, toxicity checks were continued until new toxicity event or last clinic date. In the event of no posttreatment toxicity check, the date of last toxicity check was dated as day of initial SRS treatment. Patients with posttreatment MRI’s but no posttreatment toxicity checks were included in the treatment failure analysis but not the toxicity analysis.

Overall local control and freedom from toxicity were estimated using the Kaplan–Meier method and were measured from initial SRS treatment date. Living patients were censored at time of most recent clinical encounter or most recent diagnostic MRI or CT. Kaplan–Meier estimates between the groups were compared using the log-rank test. Differences in patient characteristics were calculated using Mann-Whitney and Pearson chi-squared tests. The effect of patient, tumor, and treatment parameters on local control and toxicity were assessed using univariate and multivariate Cox proportional hazard models. Variables with *P* value less than 0.2 on univariate analysis were included in the multivariate analysis. All statistical tests were performed with SPSS software (IBM SPSS version 29.0).

## Results

### Patient and treatment characteristics

A total of 387 patients and 1734 tumors were identified that received either 9 Gy × 3 fx or 6 Gy × 5 fx in our department from 2015 to 2022. A total of 519 tumors were excluded including 62 who received prior treatment (surgery or SRS) to the lesion of interest, 75 who were treated with preop or postop intent, and 382 who did not have posttreatment imaging. A total of 1215 tumors from 251 patients remained that met inclusion criteria for this analysis. Patient and treatment characteristics are presented in Table 1. Tumor size characteristics are displayed in Table 2.

**Table 1:** Summary of patient characteristics

	**Total**	9 Gy × 3 fx	6 Gy × 5 fx	*P* value
Number of tumors	1215	256	959	
Age at the time of SRS (mean)	60 (27-90)	63 (28-90)	60 (27-90)	
Race:				.013
White	954	215 (84%)	739 (77%)	
Black	213	40 (16%)	173 (18%)	
Asian	15	1 (0.4%)	14 (1.5%)	
Hispanic	5	0	5 (.5%)	
Other	28	0	28 (3%)	
Gender				<.001
Male	550	161 (63%)	389 (41%)	
Female	665	95 (37%)	570 (59%)	
Histology				<.001
Lung	375	47 (18%)	328 (34%)	
Melanoma	267	42 (16%)	225 (24%)	
Breast	246	55 (22%)	191 (20%)	
Renal	84	36 (14%)	48 (5%)	
Colorectal	29	0	29 (3%)	
Other	179	61 (24%)	118 (12%)	
Unknown	35	15 (6%)	20 (2%)	
Immunotherapy				<.001
Yes	480	136 (53%)	344 (40%)	
No	684	115 (45%)	569 (56%)	
Unknown	51	5 (2%)	46 (4%)	
Tumor diameter (mean cm)	1.22 (0.20-4.39)	0.99 (0.20-3.17)	1.28 (0.23-4.39)	<.001
Median tumor diameter (cm)	0.91	0.77	0.98	<.001
Distance from isocenter (mean cm)	4.71 (0-13.42)	4.87 (0.05-9.99)	4.67 (0-13.42)	0.154
Months of clinical follow-up	13 (0-94)	12 (0-51)	14 (0-94)	<.001
Months of MRI follow-up	13 (0-94)	11 (0-51)	13 (0-94)	.002

Continuous variable *P* value calculated using the Mann-Whitney *U* test for nonparametric variables. Categorical *P* values calculated using Pearson chi-squared test

**Table 2:** Tumor numbers by size and dose schedule

	**Total**	9 Gy × 3 fx	6 Gy × 5 fx
Number of tumors	1215	256	959
<2 cm	977	226	751
2-4 cm	230	30	200
>4 cm	16	8	8

### Local control outcomes

**Figure 1:**
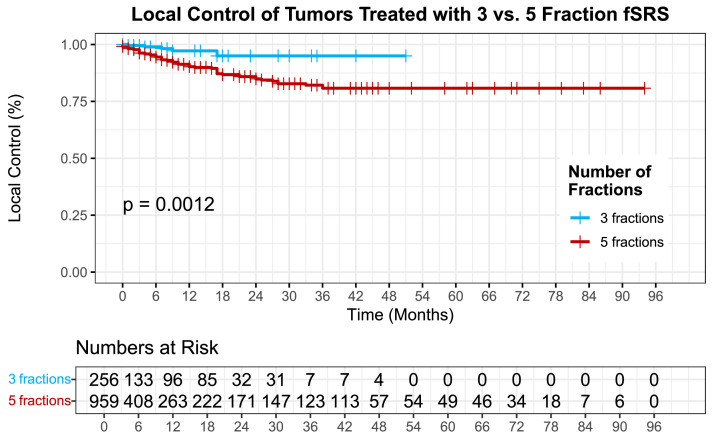
Local control of tumors treated with 3 vs. 5 fraction fSRS (*P* = .001). *Abbreviation:* fSRS = fractionated stereotactic radiosurgery.

At 12 months, 358 (29%) tumors that had not failed or had toxicity remained evaluable with radiographic and clinical follow-up. Mean clinical and radiographic follow-up was 13 months (range, 0-94) for both. 12- and 24-month local control was 93% (±0.010) and 88% (±0.015) for the whole population. When stratified by dosing schedule, a 12-month local control was 97% (± 0.014) and 91% (± 0.013) for 3 fx and 5 fx, respectively (*P* = .001) (Fig. 1). A 24-month local control of tumors treated 3 fx vs 5 fx was 95% (± 0.021) and 86% (± 0.018), respectively (*P* = .001).

**Figure 2:**
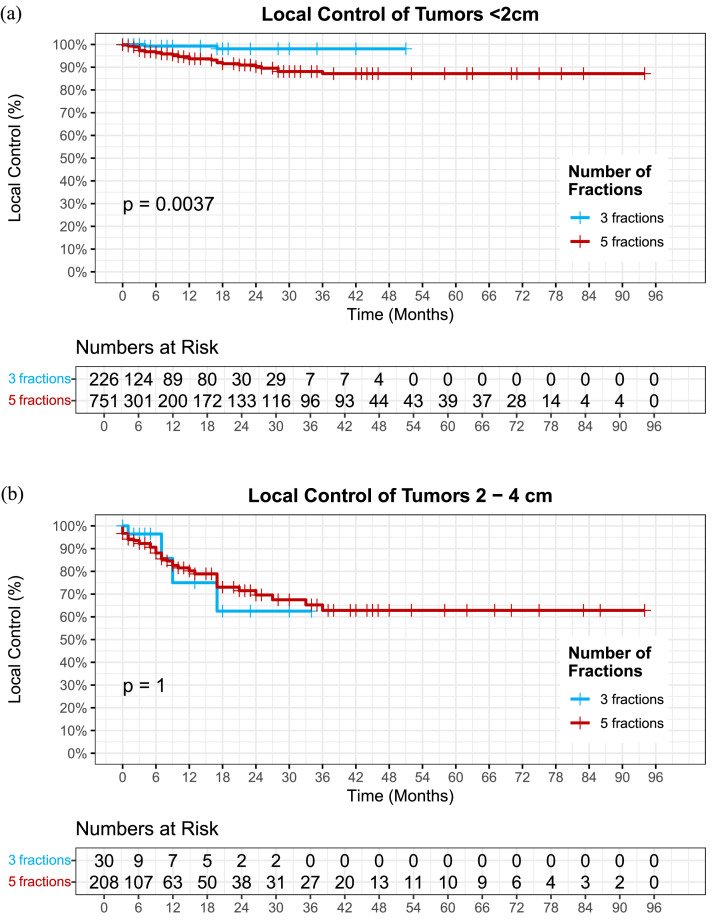
(A) Local control among tumors <2cm by fraction number (*P* = .004). (B) Local control among tumors 2-4 cm by fraction number (*P* = .994).

Tumors were then stratified by size categories (<2 cm, 2-4 cm, or >4 cm). Tumors <2 cm had significantly improved 1-year tumor control with 3 fx regimen compared with 5 fx regimen at 99% (±0.007) vs. 95% (±0.0012) (*P* = 0.004) (Fig. 2a). There was no statistically significant difference in local control between the 3 fx and 5 fx cohort among tumors 2-4 cm (*P* = .994) (Fig. 2b) or tumors >4 cm (number of events were too small for statistical analysis).

Table 3 presents the results of the multivariate Cox proportional hazard models for potential predictors of local failure. Four variables had significance in the univariate Cox regression and were included in the multivariate analysis. Five fraction regimen (hazard ratio, 3.02; *P* = .012), tumor size (hazard ratio, 1.05; *P* < .001), and histology (*P* = .003) were the only factors that retained statistical significance as independent predictors of local control. Increased tumor volume, 5 fraction treatment regimen, melanoma, and colorectal histology were associated with worse local control.

**Table 3:** Cox multivariate regression for tumor control

	*P* value	HR	CI
**Tumor histology**			
Lung	.003		
Melanoma	.018	2.07	1.1-3.8
Breast	.070	1.79	1.0-3.4
Renal	.672	.64	.1-4.9
Colorectal	.000	6.56	2.4-18.0
Other	.693	.82	.3-2.2
Unknown	.056	3.35	1.0-11.6
**3 vs. 5 fractions**	.012	3.02	1.3-7.1
**Tumor size (cc)**	.000	1.05	1.0-1.1
**Distance from isocenter (cm)**	.249	.99	1.0-1.0

*Abbreviations:* HR = hazard ratio; CI = confidence interval.

### Toxicity

**Figure 3:**
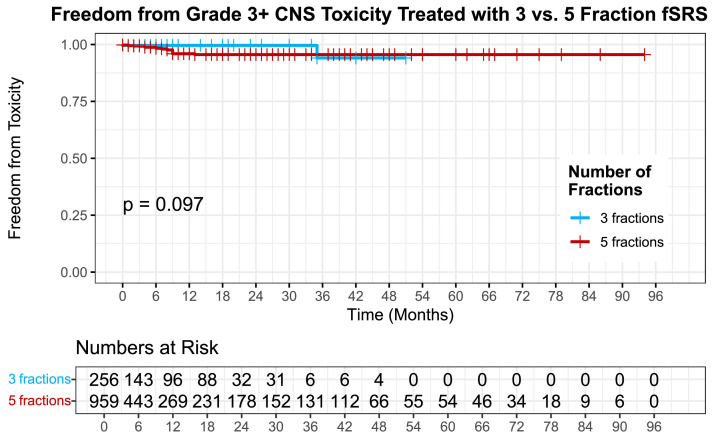
Freedom from toxicity of tumors treated with 3 vs. 5 fraction fSRS (*P* = .097). *Abbreviation:* fSRS = fractionated stereotactic radiosurgery.

In total, there were 18 grade 3 toxicities, 4 grade 4 toxicities, and 1 grade 5 toxicities among the whole cohort. Among the grade 3 toxicities, 7 were new onset seizures, 6 were cerebral edema requiring hospitalization, 4 were radiographically diagnosed radionecrosis, and 1 was intracerebral hemorrhage requiring hospitalization. Among grade 4 toxicities, 3 patients required urgent surgical resection with pathologically diagnosed radionecrosis and 1 for cerebral edema of unknown cause (pathology not available). Seven patients with radionecrosis were also scored as local failure because of the presence of malignancy and background extensive radionecrosis in the resected surgical specimen. The single grade-5 toxicity was for death shortly after resection with pathology showing both viable tumor and extensive background radionecrosis. Among the entire cohort, the 2-year freedom from G3+ toxicity was 97% (±0.007). Freedom from G3+ toxicity was similar between both cohorts with 1- and 2-year toxicity events of 99% (±0.004) in the 3 fx and 96% (±0.009) in the 5 fx group, respectively (*P* = .097) (Fig. 3).

## Discussion

Several dose schedules have demonstrated effective local control with acceptable toxicity risk; however, comparative studies are generally lacking.^10^, ^11^, ^12^, ^13^, ^14^ The purpose of this retrospective review was to compare 2 common fSRS regimens, 6 Gy × 5 fx and 9 Gy × 3 fx, for their effect on local control and toxicity. Treatment was well tolerated with similar rates of tumor control and toxicity compared with historical data.^10^^,^^23^ The results from this study indicated superior local control with 3 fx compared with 5 fx, particularly among small tumors (<2 cm). Additionally, the risk of toxicity was similar and low between the 2 dose schedules.

One-year local control rates among previously reported fSRS regimens range from 52 to 95%.^25^, ^26^, ^27^, ^28^, ^29^, ^30^, ^31^ Although difficult to compare due do potential differences in population, dose schedule, tumor size, and inclusion criteria, our 1-year local control rate of 93% compares favorably. Minitti et al^23^ previously reported 1-year local control of 90% among tumors >2 cm treated with 9 Gy × 3, which is similar to what was found in this present study.^23^ Our higher 1-year local control rate of 97% among tumors treated 9 Gy × 3 fx likely reflects that our study included tumors <2 cm as a single prescription was used when treating multiple brain metastases. The 1-year local control of 91% among tumors treated 6 Gy × 5 fx in this study is consistent with prior data from Marcrom et al.^10^

In this study, we found that tumors receiving 9 Gy × 3 fx had superior 1-year local control (97%) compared with 91% with tumors treated 6 Gy × 5 fx. The mean tumor diameter was 2 mm larger in the 5-fraction group compared with the 3-fraction group (0.77 cm vs. 0.98 cm) (Table 1); however, this is likely clinically insignificant. Multivariate analysis reflected several known predictors for worse tumor control including increased tumor volume as well as melanoma and colorectal histology.^32^, ^33^, ^34^ Independent of these factors, 6 Gy × 5 fraction regimen was also associated with worse tumor control compared with 9 Gy × 3. The improved local control likely reflects that 9 Gy × 3 fx (27 Gy) has a higher BED10 (51.3) than 6 Gy × 5 fx (48). Interestingly, we did not find a difference in toxicity between the 2 groups despite the BED3 difference of 108 vs. 90 for 3 fx vs. 5 fx. Freedom from grade 3 or higher toxicity was similar among both with 1- and 2-year toxicity events of 99% in the 3 fx and 96% in the 5 fx group (*P* = .097). Most of the G3+ toxicities were grade 3 because of new onset seizures or cerebral edema requiring hospitalization. There were no grade 3 or greater toxicity events because of radionecrosis in the 3 fx group.

The use of single prescription when treating multiple brain tumors has the advantage of increased efficiency with planning and reduced patient time on the table. There remain questions as to whether multiple prescriptions are preferred when treating a patient with multiple brain metastases of various sizes (single fraction SRS for small metastases and fSRS for larger metastases). Our department policy of treating different size tumors with the same number of fractions for all tumors is based on reports of acceptable tumor control and toxicity in the literature as well as this study with hypofractionated SRS.^10^^,^^35^ Although the benefit of 3 fx over 5 fx was significant throughout the entire population of this study, only tumors <2 cm remained significant when stratified by tumor size (0-2 cm, 2-4 cm, >4 cm) (*P* = .002). This suggests that the higher dose of the 3 fx regimen benefits smaller tumors (<2 cm) while having little effect on tumors 2-4 cm. Of note, there were only 2 tumors >4 cm treated with the 9 Gy × 3 fx, limiting comparability. Future studies should compare smaller tumors treated single fraction vs. 3 fx to determine if separate dose prescriptions based on tumor size offsets increased planning and treatment delivery time.

This study was retrospective and therefore has inherent limitations including nonrandomization of dose schedule. The limitations were mitigated to the best of our ability through well-defined inclusion and exclusion criteria. The median follow-up of this study was only 4 months; however, 358 and 202 tumors remained evaluable at 12 and 24 months, respectively. The sharp reduction in evaluable tumors at 1 year likely reflects the morbid nature of metastatic cancers. This study also included patients with recurrent disease if they exhibited evidence of distant brain failure. Another limitation is the lack of data regarding nonimmunotherapy systemic therapies such as anaplastic lymphoma kinase or epidermal growth factor receptor inhibitors, which may have affected local control. Despite the modest median follow-up, the large number of evaluable tumors at 12 and 24 months still render the data in this review useful.

## Conclusion

The results of this study suggest that both dose schedules are associated with high rates of local control and acceptable toxicity rates. In this series where brain tumors were all treated with single prescription regardless of size, patients receiving 9 Gy × 3 fx for brain metastases had higher overall tumor control and similar toxicities compared with tumors treated 6 Gy ×5 fx. When stratified by tumor size, local control benefit from 9 Gy × 3 fx was most significant among tumors <2 cm. Increased tumor size, melanoma, and colorectal histology were also found to be independent predictors of worse local control in this study. As 9 Gy × 3 fx allows for decreased treatment days, higher local control, and similar toxicity rates, it should be the preferred regimen over 6 Gy × 5 fx when treating multiple tumors <4 cm. Future studies are needed for tumors >4 cm as well as comparison studies of tumors <2 cm treated single fraction vs. fSRS. Additionally, future directions should include neurocognitive or quality of life data as this is important when evaluating for candidacy of radiosurgery.

## Disclosures

John Fiveash reports a relationship with Varian Medical Systems Inc that includes research and educational grants. Richard Popple reports a relationship with Varian Medical Systems Inc that includes consulting or advisory and travel reimbursement. Drexel Boggs reports a relationship with Varian Medical Systems Inc that includes consulting or advisory and travel reimbursement. Christopher Willey reports a relationship with Varian Medical Systems Inc that includes consulting or advisory and travel reimbursement. If there are other authors, they declare that they have no known competing financial interests or personal relationships that could have appeared to influence the work reported in this paper.
